# Transjugular LuX-Valve Plus transcatheter tricuspid valve replacement for torrential tricuspid regurgitation in a patient with a giant right atrium and multiple prior valve interventions: a case report

**DOI:** 10.1093/ehjcr/ytag658

**Published:** 2026-09-02

**Authors:** Quntai Qiu, Xinlei Wu, Daozhu Wu, Xinmin Zhang, Lianpin Wu

**Affiliations:** Zhejiang-Ireland Joint Laboratory for Precision Diagnosis and Treatment of Valvular Heart Diseases, The Second Affiliated Hospital of Wenzhou Medical University, 1111 East Wenzhou Avenue, Longwan, Wenzhou 325024, China; Department of Cardiology, The Second Affiliated Hospital and Yuying Children's Hospital of Wenzhou Medical University, 1111 East Wenzhou Avenue, Longwan, Wenzhou 325024, China; Zhejiang-Ireland Joint Laboratory for Precision Diagnosis and Treatment of Valvular Heart Diseases, The Second Affiliated Hospital of Wenzhou Medical University, 1111 East Wenzhou Avenue, Longwan, Wenzhou 325024, China; Department of Cardiology, The Second Affiliated Hospital and Yuying Children's Hospital of Wenzhou Medical University, 1111 East Wenzhou Avenue, Longwan, Wenzhou 325024, China; Zhejiang-Ireland Joint Laboratory for Precision Diagnosis and Treatment of Valvular Heart Diseases, The Second Affiliated Hospital of Wenzhou Medical University, 1111 East Wenzhou Avenue, Longwan, Wenzhou 325024, China; Department of Cardiology, The Second Affiliated Hospital and Yuying Children's Hospital of Wenzhou Medical University, 1111 East Wenzhou Avenue, Longwan, Wenzhou 325024, China; Zhejiang-Ireland Joint Laboratory for Precision Diagnosis and Treatment of Valvular Heart Diseases, The Second Affiliated Hospital of Wenzhou Medical University, 1111 East Wenzhou Avenue, Longwan, Wenzhou 325024, China; Department of Cardiology, The Second Affiliated Hospital and Yuying Children's Hospital of Wenzhou Medical University, 1111 East Wenzhou Avenue, Longwan, Wenzhou 325024, China; Zhejiang-Ireland Joint Laboratory for Precision Diagnosis and Treatment of Valvular Heart Diseases, The Second Affiliated Hospital of Wenzhou Medical University, 1111 East Wenzhou Avenue, Longwan, Wenzhou 325024, China; Department of Cardiology, The Second Affiliated Hospital and Yuying Children's Hospital of Wenzhou Medical University, 1111 East Wenzhou Avenue, Longwan, Wenzhou 325024, China

**Keywords:** Tricuspid regurgitation, Transcatheter tricuspid valve replacement, LuX-Valve Plus, Giant right atrium, Case report

## Abstract

**Background:**

Transcatheter tricuspid valve replacement (TTVR) is an option for selected patients with severe tricuspid regurgitation (TR) who are at high surgical risk, but experience remains limited in those with extreme right-heart anatomy, including a giant right atrium and a markedly enlarged elliptical tricuspid annulus.

**Case summary:**

A 62-year-old man was admitted with progressive dyspnoea and lower-extremity oedema (NYHA class IV). He had previously undergone mechanical mitral valve replacement and transcatheter aortic valve replacement. Echocardiography and computed tomography angiography (CTA) showed torrential TR, a giant right atrium (11.59 × 7.73 cm), preserved right ventricular systolic function, and a markedly elliptical annulus with a perimeter-derived diameter of 55.5 mm. Persistent TR despite satisfactory left-sided prosthetic valve function prompted transcatheter evaluation. Redo surgery was prohibitive and transcatheter edge-to-edge repair was unsuitable. The Heart Team selected LuX-Valve Plus because its leaflet graspers and septal anchor provide radial force-independent multipoint fixation. A 79.2-mm bend-point-to-annulus distance exceeded the proposed 45–70 mm range, requiring a slightly lateral jugular entry angle and complicating depth control and coaxial alignment. The mechanical mitral prosthesis limited visualization by transoesophageal echocardiography (TEE) but served as a fluoroscopic landmark; TEE and fluoroscopy enabled successful implantation. At 1 month, NYHA class improved to II, with stable prosthetic haemodynamics, no obvious transvalvular regurgitation, and trivial paravalvular leak; findings remained stable at 6–7 weeks.

**Discussion:**

This case highlights the feasibility of transjugular LuX-Valve Plus TTVR in extreme right-heart anatomy and emphasizes the importance of anatomy-driven device selection, tailored delivery and imaging strategies, and individualized postprocedural management.

Learning points

- An out-of-range bend-point-to-annulus distance may be managed by tailoring the transjugular delivery trajectory.
- A mechanical mitral prosthesis may hinder TEE visualization while simultaneously serving as a fluoroscopic landmark.

## Introduction

Severe tricuspid regurgitation (TR) is associated with right-sided heart failure, recurrent hospitalization, and increased mortality.^1^ Transcatheter tricuspid interventions offer a less invasive therapeutic alternative for patients at high or prohibitive surgical risk, but marked annular dilatation and large coaptation gaps may preclude transcatheter edge-to-edge repair (TEER).^2,3^ Experience with transcatheter tricuspid valve replacement (TTVR) in patients with a giant right atrium (GRA) and prior multivalvular interventions remains limited.^4,5^ We report such a patient who underwent transjugular LuX-Valve Plus TTVR, focusing on device selection, procedural adaptation, and early postprocedural management.

## Summary figure

**Figure:**
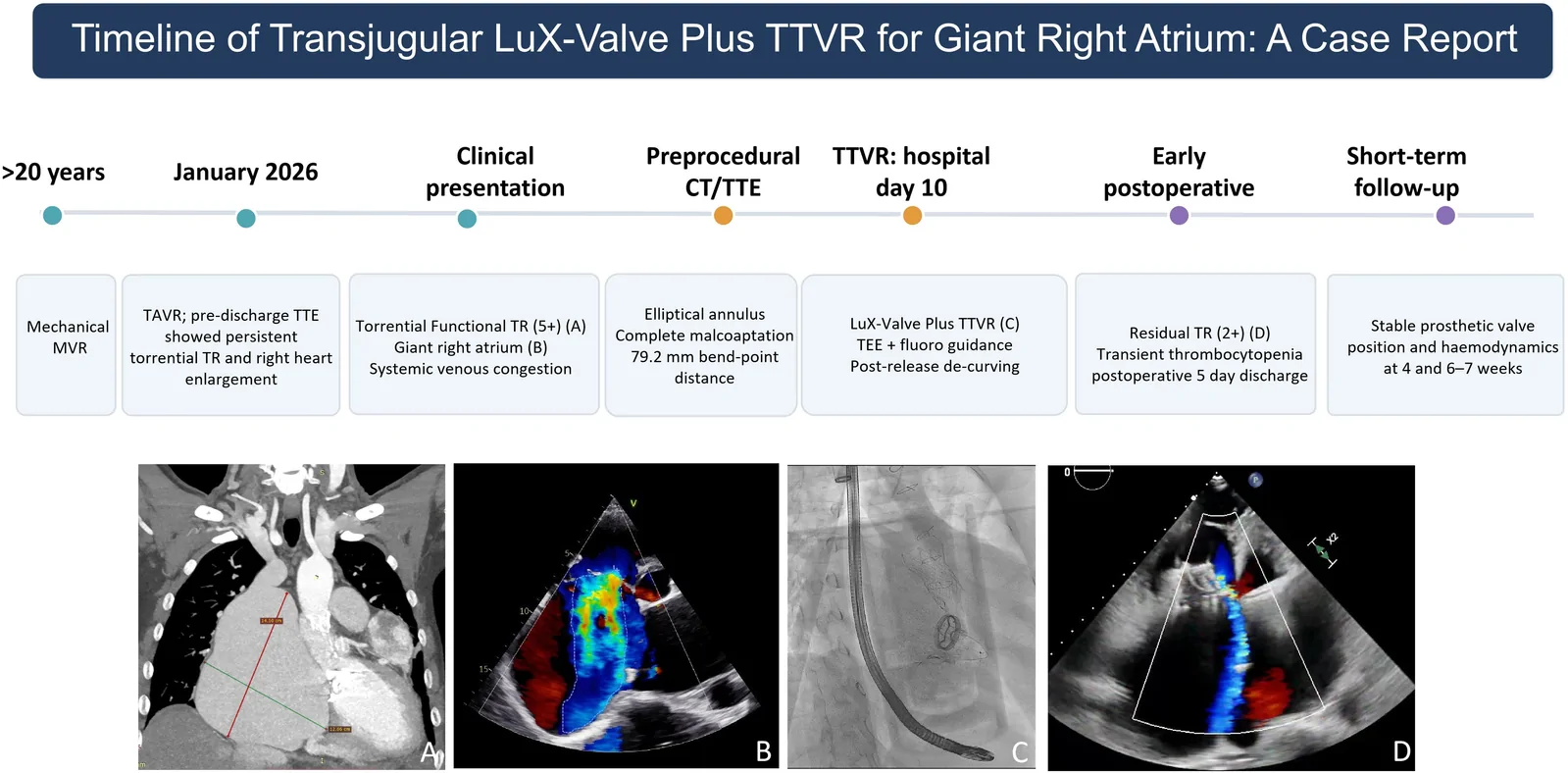


## Case presentation

A 62-year-old man was admitted with progressive dyspnoea and bilateral pitting lower-extremity oedema for over 2 weeks (NYHA class IV). He had undergone surgical mitral valve replacement with a mechanical prosthesis over 20 years earlier and transcatheter aortic valve replacement with a TaurusMax® valve (Peijia Medical Technology, Suzhou, China) in January 2026. He also had persistent atrial fibrillation, hypertension, and type 2 diabetes and was receiving long-term warfarin therapy. Laboratory testing showed haemoglobin 92 g/L, platelet count 73 × 10^9^/L, NT-proBNP 1570 pg/mL, creatinine 96 μmol/L (eGFR 77 mL/min/1.73 m^2^), total/direct bilirubin 23.9/7.2 μmol/L, and GGT 76 U/L.

**Figure 1:**
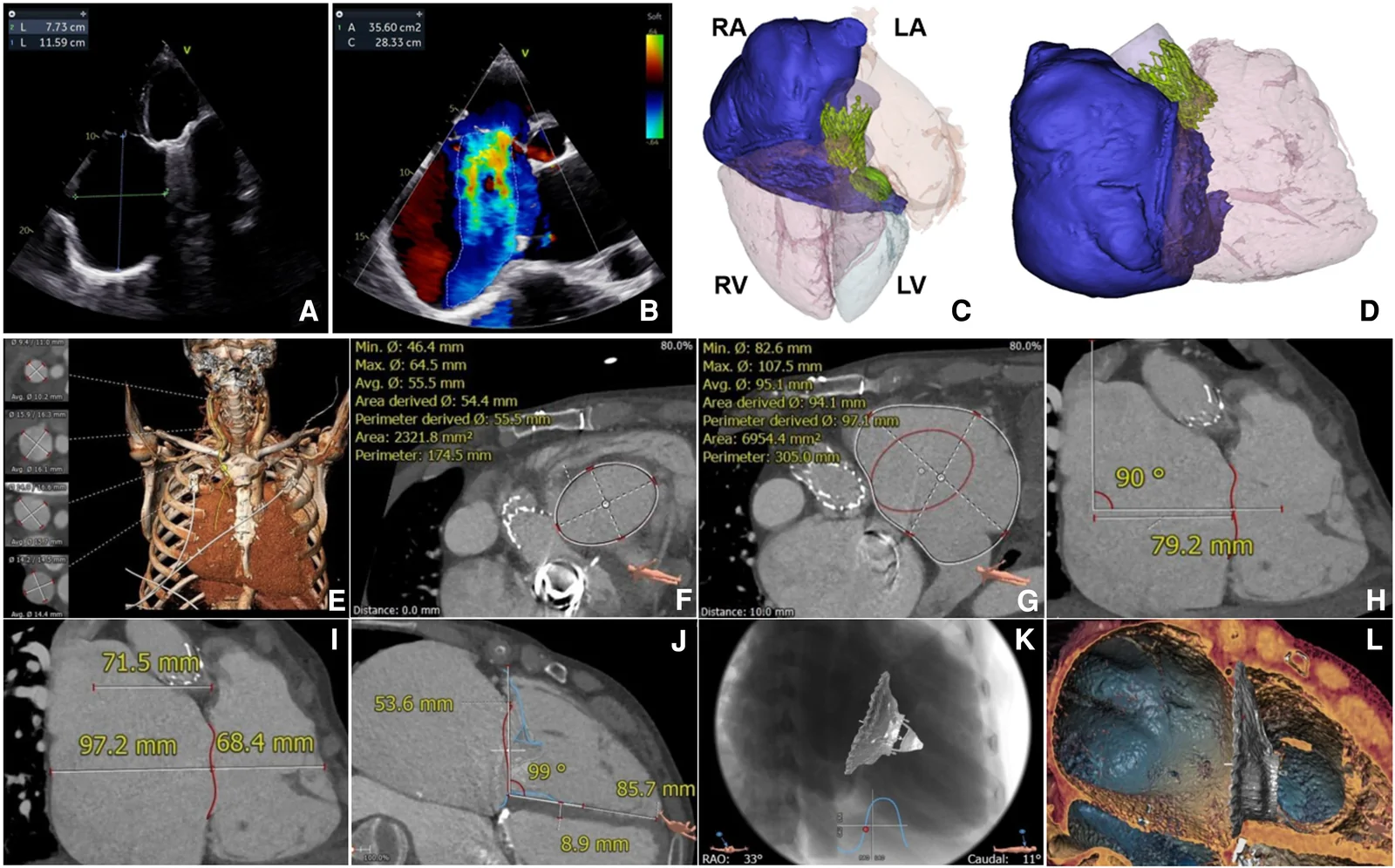
Preprocedural multimodality imaging and CTA-based procedural planning. (*A*, *B*) TTE showed marked right atrial enlargement (11.59 × 7.73 cm) and torrential tricuspid regurgitation with a colour Doppler jet area of 35.6 cm^2^. (*C*, *D*) 3D CTA reconstruction demonstrated a giant right atrium and distorted right-heart geometry. (*E–G*) CTA-based planning assessed the transjugular access route, annular dimensions, and dimensions 10 mm above the annular plane. (*H–L*) Additional measurements included the planned bend-point-to-annulus distance (79.2 mm), right ventricular working length, annulus–septal angle, septal thickness, and fluoroscopic deployment projection.

Preprocedural transthoracic echocardiography (TTE) showed a GRA (11.59 × 7.73 cm), torrential TR with complete leaflet malcoaptation, and a 55-mm annulus (*Figure 1A* and *B*); vena contracta width was 15 mm, and jet area was 35.6 cm^2^. Right ventricular systolic function was preserved (tricuspid annular plane systolic excursion 23 mm, S′ 16 cm/s, and right ventricular fractional area change 46.4%), and estimated pulmonary artery systolic pressure was 46 mmHg. Left ventricular end-diastolic diameter/left ventricular end-systolic diameter were 63/46 mm, and left ventricular ejection fraction was 52%. The mechanical mitral prosthesis and transcatheter aortic prosthesis functioned satisfactorily, with mean gradients of 4 and 6 mmHg, respectively, and no paravalvular leak. 3D computed tomography angiography (CTA) reconstruction confirmed the GRA and distorted right-heart geometry (*Figure 1C* and *D*). Persistent torrential TR (5+) and systemic venous congestion despite satisfactory left-sided prosthetic valve function prompted evaluation for transcatheter tricuspid intervention.

The Heart Team considered redo surgery prohibitive (TRI-SCORE 6/12; predicted in-hospital mortality 22%)^6^ and TEER anatomically unsuitable.^3^ Dedicated CTA-based planning confirmed a feasible transjugular route and a markedly elliptical annulus with a perimeter-derived diameter of 55.5 mm; the perimeter-derived diameter at a plane 10 mm above the annulus was 97.1 mm (*Figure 1E–L*). The planned bend-point-to-annulus distance, defined as the distance from the hypothetical delivery-system bend point to the tricuspid annular centre, was 79.2 mm, exceeding the proposed 45–70 mm range.^7^ The markedly elliptical annulus was considered unfavourable for radial force-dependent systems as secure fixation could be challenging.^5,7,8^ A LuX-Valve Plus® (JS/TTVI-30-60; Jenscare Biotechnology Co., Ltd., Ningbo, China) was selected because its leaflet graspers and interventricular septal anchor provide radial force-independent multipoint fixation.^5,7,9,10^ Before TTVR, he remained stable on room air with oral diuretics only, without intravenous diuretics or inotropes. Warfarin was withheld 24 h before TTVR without bridging.

**Figure 2:**
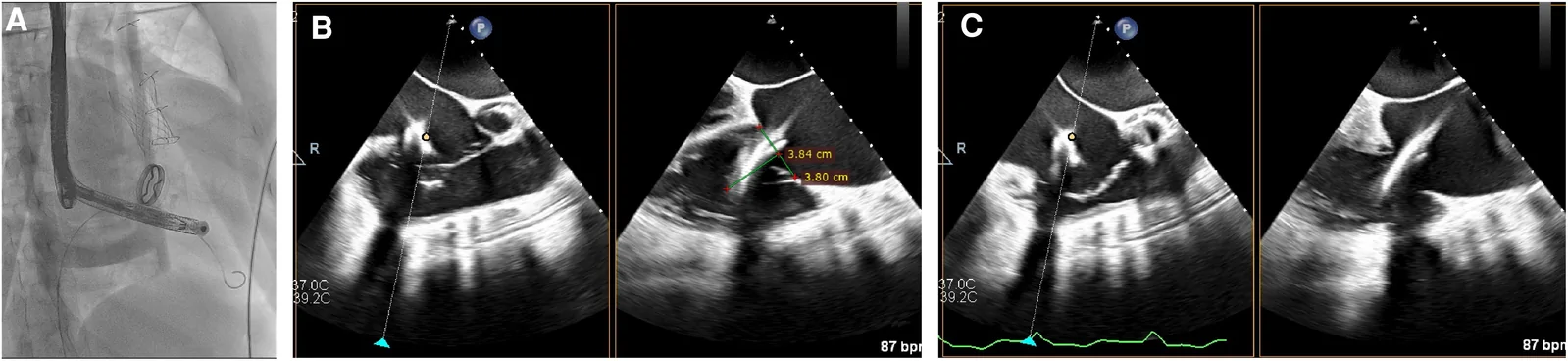
Intraprocedural multimodality imaging during transjugular LuX-Valve Plus deployment. (*A*) Fluoroscopy showed the delivery-system trajectory; the radiopaque mechanical mitral prosthetic frame served as a landmark for annular localization and system orientation. (*B*, *C*) Transoesophageal echocardiography showed the delivery system–annulus relationship for depth assessment and confirmation of coaxial alignment.

TTVR was performed on hospital day 10 via the right internal jugular vein. Because the planned bend-point-to-annulus distance exceeded the proposed range, the delivery system was introduced at a slightly lateral angle, rather than vertically along the medial wall of the vein, to reduce the required bend. Despite this adjustment, the GRA complicated depth control and coaxial alignment. Acoustic shadowing and reverberation from the mechanical mitral prosthesis limited conventional TEE assessment of the tricuspid annular plane and delivery-system orientation. 3D TEE with multiplanar reconstruction was used to define the delivery system-annulus relationship and confirm coaxial alignment. Under fluoroscopy, the radiopaque mitral prosthetic frame served as a landmark for annular localization and system orientation (*Figure 2*; Supplementary material online, *Videos S1* and *S2*).

**Figure 3:**
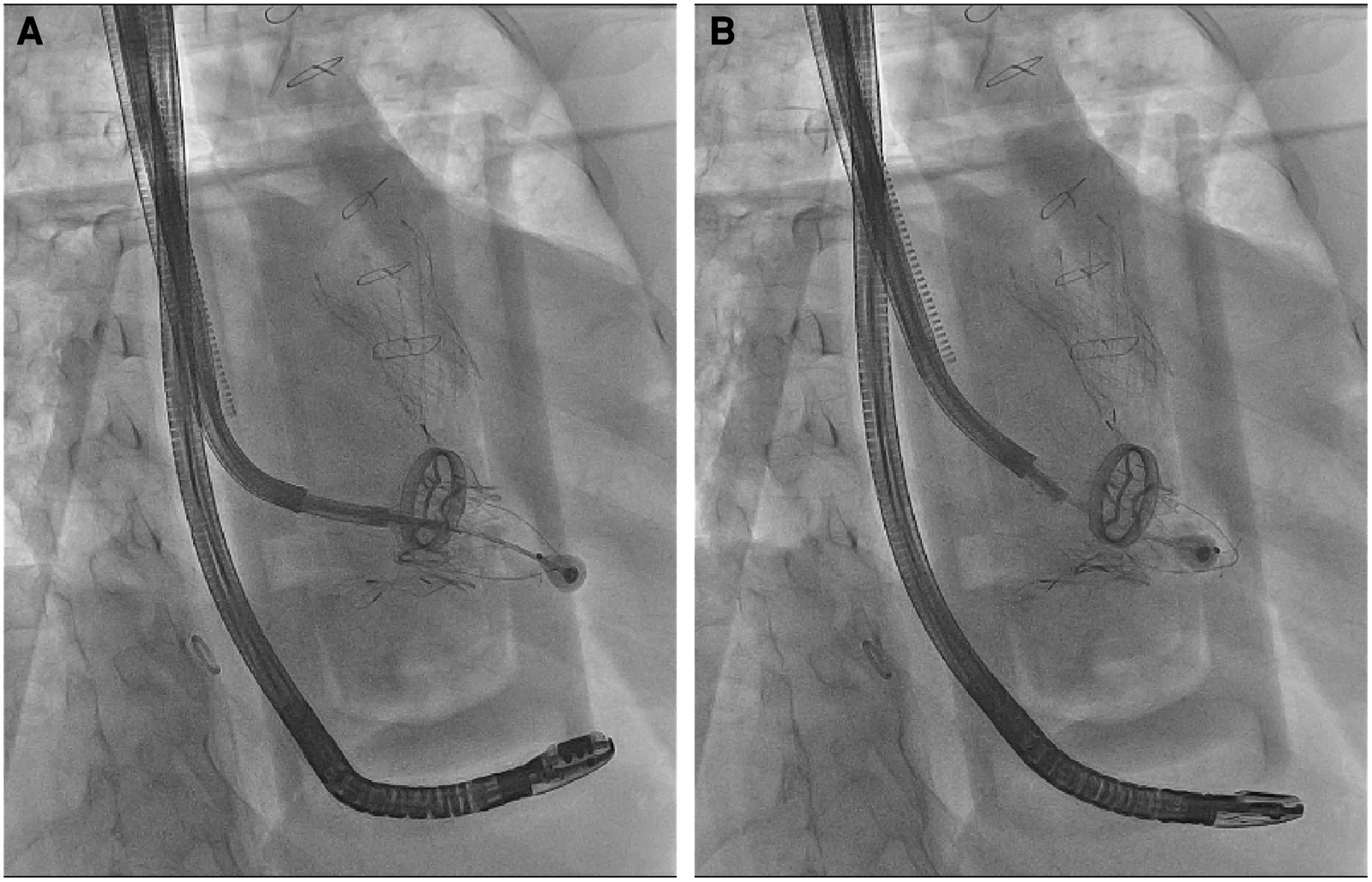
Controlled straightening of the delivery system after valve release. (*A*) Fluoroscopy showed marked delivery-system curvature after valve release. (*B*) Controlled straightening improved system conformation before withdrawal.

After satisfactory depth and coaxial alignment were achieved, the ventricular portion was unsheathed to expose the anterior and posterior leaflet graspers (‘rabbit ears’). The graspers were positioned beneath the corresponding native leaflet segments, and leaflet capture was confirmed fluoroscopically (see Supplementary material online, *Video S3*). The atrial portion was then deployed (see Supplementary material online, *Video S1*). After satisfactory alignment and apposition were confirmed, the septal anchor tube was advanced towards the interventricular septum. Fluoroscopy and TEE confirmed appropriate anchor alignment and contact of the anchor plate with the septum, after which the three anchor hooks were deployed (see Supplementary material online, *Video S2*). Following complete valve release, marked delivery-system curvature required gradual, controlled straightening before withdrawal (*Figure 3*). Final TEE confirmed satisfactory device position without obvious residual TR. Intraprocedural heparin (6000 IU) was reversed with 50 mg of protamine, after which the delivery system was removed and jugular haemostasis was achieved.

**Figure 4:**
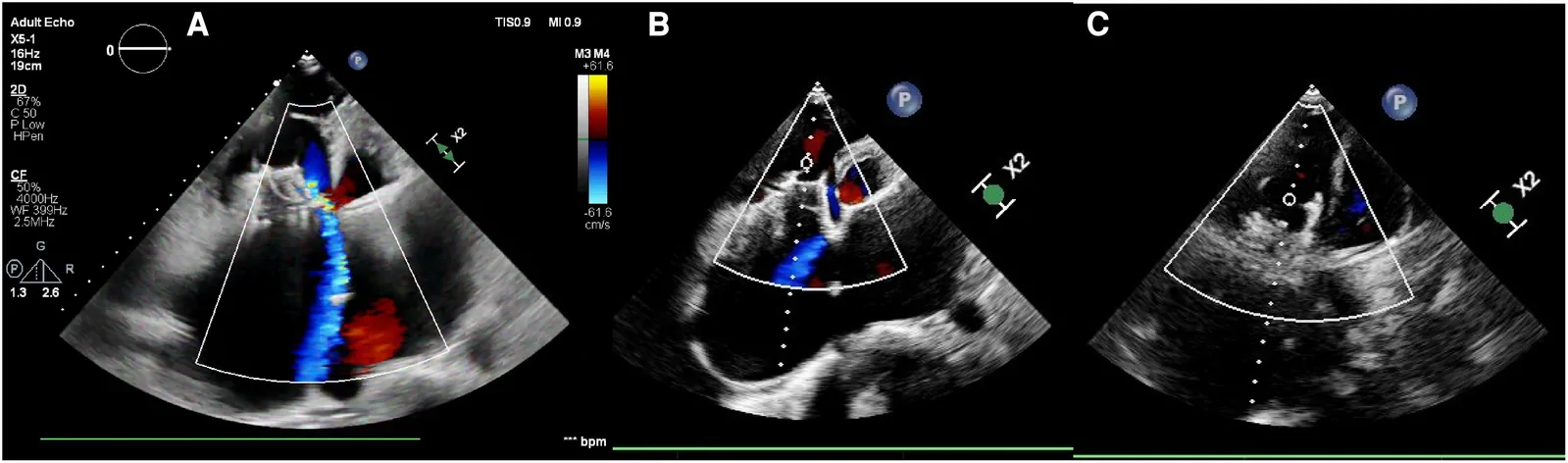
Echocardiographic follow-up after LuX-Valve Plus implantation. (*A*) Early postprocedural colour Doppler echocardiography showing moderate residual tricuspid regurgitation (2+). (*B*) At 1 month, the prosthetic position and haemodynamics were stable, with no obvious transvalvular regurgitation and only trivial paravalvular leak. (*C*) At 6–7 weeks, the prosthetic position and haemodynamics remained stable, with no obvious transvalvular regurgitation or paravalvular leak.

Postoperatively, the patient was monitored in the cardiac care unit and transferred to the general ward the following day. The platelet count declined from an already low baseline to 44 × 10^9^/L and partially recovered after platelet transfusion. Early bedside echocardiography showed the prosthesis in position with moderate residual TR (2+) (*Figure 4A*). Postprocedural D-dimer was elevated, and right internal jugular ultrasound could not exclude thrombus or intramural haematoma; warfarin was therefore temporarily replaced with low-molecular-weight heparin under close monitoring. Five days after TTVR, he was discharged with improved dyspnoea and oedema; NT-proBNP was 6360 pg/mL. Warfarin was resumed at 1.875 mg once daily, with furosemide and spironolactone prescribed at 20 mg once daily each. At 1 month, NYHA class had improved to II and NT-proBNP had decreased to 3480 pg/mL, although residual oedema persisted. Echocardiography showed stable prosthetic haemodynamics (peak velocity 1.6 m/s; mean gradient 4 mmHg), no obvious transvalvular regurgitation, and trivial paravalvular leak. At 6–7 weeks, prosthetic haemodynamics remained stable (peak velocity 1.3 m/s; mean gradient 4 mmHg), with no obvious transvalvular regurgitation or paravalvular leak (*Figure 4B*, *C*).

## Discussion

This case highlights three key considerations for transjugular TTVR in extreme right-heart anatomy: anatomy-driven device selection, adaptation of delivery and imaging strategies, and individualized postprocedural management.

The LuX-Valve Plus offers a broad size range and uses leaflet graspers and a septal anchor for radial force-independent multipoint fixation.^7,10^ In an international registry of 76 patients, 75% received devices ≥55 mm, with no significant differences in procedural outcomes or adverse events between device-size groups.^11^ Accordingly, the LuX-Valve Plus was considered a reasonable option for this patient.

Even after anatomy-driven device selection, procedural success depended on adapting the delivery strategy to the patient’s right-heart geometry. The planned bend-point-to-annulus distance exceeded the proposed 45–70 mm range, prompting a slightly lateral jugular entry angle to reduce the required bend. In this patient, the out-of-range parameter did not preclude treatment but required modification of the delivery trajectory. The pre-existing mechanical mitral prosthesis was both an echocardiographic obstacle and a fluoroscopic aid. Acoustic shadowing and reverberation impaired TEE visualization of the tricuspid annular plane, whereas the radiopaque frame provided a fixed fluoroscopic landmark for annular localization and system orientation.

Transient thrombocytopenia has been reported after LuX-Valve Plus implantation, and the further platelet decline in this patient with baseline thrombocytopenia supports close monitoring.^7^ Concurrent bleeding and thrombotic concerns prompted temporary anticoagulation adjustment under close surveillance. Serial echocardiography confirmed stable short-term prosthetic haemodynamics, with no obvious transvalvular regurgitation and no more than trivial paravalvular leak at follow-up. This case supports the feasibility of transjugular LuX-Valve Plus implantation in extreme right-heart anatomy, although larger multicentre studies are needed to evaluate procedural safety and long-term durability.

## Lead author biography


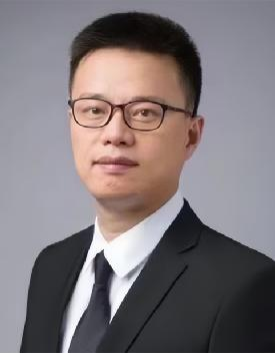
 In 2015, Lianpin Wu became a professor at Wenzhou Medical University. In 2018, he served as the academic leader of the Department of Cardiology at the Second Affiliated Hospital of Wenzhou Medical University. In 2023, he was appointed as the director of the Zhejiang-Ireland International Joint Laboratory for Precision Diagnosis and Treatment of Heart Valve Disease.

## Supplementary Material

ytag658_Supplementary_Data

## Data Availability

The data underlying this article will be shared on reasonable request to the corresponding author.
